# Pulmonary Embolism With Underlying Family History Presenting as Syncope: A Case Report

**DOI:** 10.7759/cureus.30448

**Published:** 2022-10-18

**Authors:** Amruta Chavhan, Ashish Anjankar

**Affiliations:** 1 Medicine and Surgery, Jawaharlal Nehru Medical College, Wardha, IND; 2 Biochemistry, Jawaharlal Nehru Medical College, Wardha, IND

**Keywords:** case report, genetic history, syncope, pleuritic chest pain, pulmonary embolism

## Abstract

An artery blockage by a foreign body, such as a blood clot/fat/air/cholesterol/amniotic fluid, is called an embolism. The most serious conditions caused by an embolism are stroke and pulmonary embolism. Pulmonary embolism (PE) occurs when a foreign body blocks the vessel that carries blood from the heart to the lungs. Deep vein thrombosis (DVT) has the potential to embolize and travel through the right side of the heart and become lodged in the blood-supplying artery of the lungs: the pulmonary artery. DVT is one of the major causes of pulmonary embolism. Pulmonary embolism is a life-threatening disease that can sometimes be problematic to point at, especially when the patient has no obvious symptoms. The risk factors may not be strikingly palpable, and there may also be an intersection between the symptoms and signs of pulmonary embolism and other diseases. Syncope is a comparatively easy clinical symptom to detect but has varied etiologies that lead to a standard cause in only 58% of syncopal events. It is a difficult correlation to make when syncope is the presenting symptom of pulmonary embolism. Family history in the case of undiagnosed pulmonary embolism presenting with symptoms that point in no particular direction becomes crucial in determining the disease.

## Introduction

A transient cessation of blood flow to the brain is what causes syncope. It can be brought on by a drop in blood pressure, heart rate, or variations in the distribution of blood throughout the body. Syncope is a clinical symptom that is simple to identify, but it can have a wide range of etiologies, making it difficult to associate it with a known pulmonary embolism (PE) pathology. PE can manifest in many different ways; it can range from being asymptomatic to sudden cardiac arrest. Patients with a self-reported lineage of venous thromboembolism in first-degree kin are more inclined to be diagnosed with an acute pulmonary embolism in the emergency room, even among people assumed to have a greater risk of PE.

Kelly et al. found that 19.4% of the 3024 research participants had a family history of venous thromboembolism, and 1.9% of them received an acute PE diagnosis in the emergency department. 3.2% vs. 1.6% (p = 0.009) of patients with a family history of venous thromboembolism had a PE diagnosis. 82.3% of patients tested positive for the criterion for ruling out pulmonary embolism (PERC), and 3.6% vs. 1.9% of PERC-positive patients with a family history of VTE were found to have PE (p = 0.016). PE diagnoses were more frequent among patients having a history of VTE in their family: 9.4% vs. 4.9% (p = 0.032) of patients who underwent testing for PE (3.7%) [1]. Undiagnosed PE can be life-threatening, with up to 30% of deaths among those undiagnosed.

## Case presentation

Patient information

A 38-year-old young man was brought to the Critical Care Centre by a relative due to loss of consciousness and a history of a fall 30 minutes before being admitted. The family has a history of hypertension. The patient's twin brother died due to a stroke three months ago. The patient was not on any medications before being admitted to the hospital.

Clinical findings

On clinical examination, the patient was found to be tachypneic, spO2 was 35%, blood pressure recorded was 90/70 mm Hg, respiratory rate 46 bpm, breath sounds were decreased in both the lungs and the pleural rub was noted in the right lung.

Diagnostic assessment

A two-dimensional echo revealed minimal pericardial effusion, right ventricular dilatation, and inferior vena cava congestion; no regional wall motion abnormalities were seen. Chest X-ray revealed pulmonary effusion with fistural extension; computed tomography (CT) pulmonary angiogram showed the complete collapse of the right lower lobe sparing anterior segment and right pleural effusion; partially obstructing intra-luminal thrombi in the second and third-degree branches in the postero basal segment of lower lobes; the left lung appeared normal in volume, heart and other structures were normal in size.

CBC revealed haemoglobin (Hb) was low at35 9.3 gm/dl (13-1and 8), total leukocyte count (TLC) elevated: 12400/cu.mm (4000-11000), Lymphocytes 80%, Neutrophils 20%, RBCs 18-20/HPF, protein 5.3gm/dl, glucose 182.4 mg/dl31. Peripheral smear examination showed microcytic hypochromic RBCs with mild anisopoikilocytosis, few target cells, and teardrop cells. D-dimer test came out to be 14.4 ug/mL.

**Figure 1:**
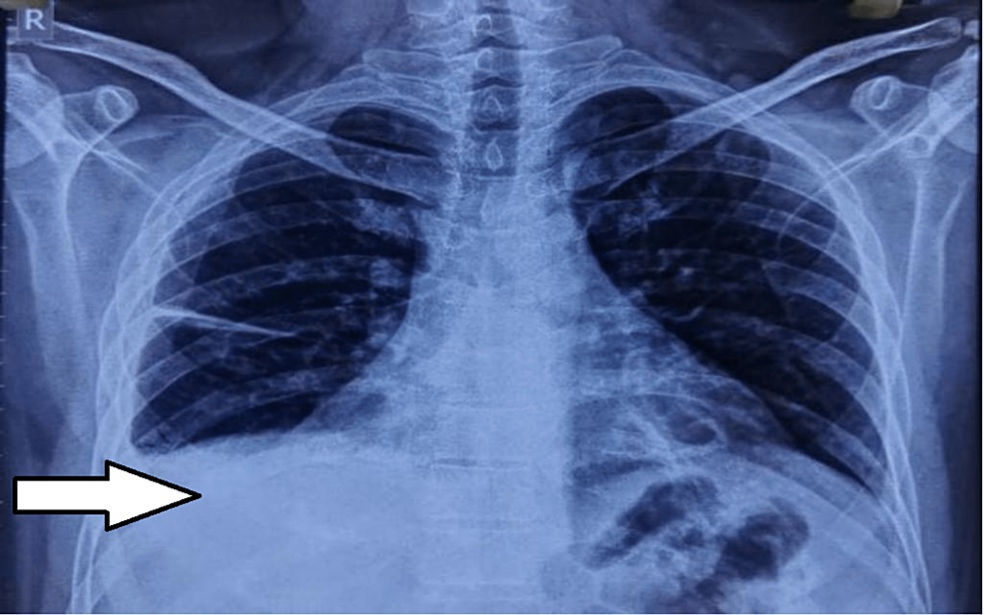
Right-sided pleural effusion

Findings of ultrasonography-guided pleural tapping confirmed right-sided pleural effusion with the sub-segmental collapse of the underlying lung (Figure 1). On pleural tapping, 300 ml of straw-colored fluid was aspirated from the right hemi thorax. A pleural fluid examination of the aspirated fluid showed pleural fluid Adenosine Deaminase (ADA) 49.5U/L (<30U/L). ECG showed sinus tachycardia with a short PR interval. C-reactive protein was elevated at 45.7 mg/L (≤5), liver function test showed elevated serum glutamic oxaloacetic transaminase (SGOT) 101U/L (up to 40), serum glutamic pyruvic transaminase (SGPT) 77.8 U/L (up to 40). The Renal Function Test came out to be normal.

The patient had no history of lower limb fracture, air travel, cancer, or hemoptysis during the preceding four weeks.

Diagnosis

This case was identified as a Pulmonary Embolism based on the recommendations from the ESC (European Society of Cardiology) and ERS (European Respiratory Society) guidelines of 2019.

## Discussion

A blood clot (thrombus) that gets dislodged in a lung artery and limits or blocks blood flow to the lung is called a pulmonary embolism (PE). Typically, a thrombus that starts in the deep venous system of the lower extremities causes a pulmonary embolism. Large thrombi that have reached the lung may lodge at the pulmonary artery's major bifurcation or its lobar branches, impairing hemodynamics.

The size and frequency of pulmonary emboli can vary greatly, and there are several unique underlying illnesses, including cancer, trauma, and a lack of protein C or S [2]. The typical triad of pleuritic chest pain, dyspnea, and hemoptysis are rare since only 11% of validated cases of pulmonary embolism in people without an underlying cardiac condition had clinically discernible DVT [3].

Although the pulmonary embolism clinical picture might vary, one of three main clinical syndromes predominately manifests in individuals with acute pulmonary embolism. A submassive embolism that completely blocks a distant branch of the pulmonary circulation frequently causes pulmonary infarct syndrome. Patients who have this sickness typically experience rales, hemoptysis, pleuritic chest tightness, and unusual X-ray changes in the chest. A submassive pulmonary embolism lacking pulmonary infarction may also cause the second type of sudden, abrupt dyspnea pattern. However, the pulse oxygen levels are commonly low even if the ECG and chest X-ray readings are typically normal. The full obstruction, somewhere between 60 and 75 percent of the pulmonary circulation, results in acute cor pulmonale syndrome, the third variety. This pattern causes shock, syncope, or unexpected death in the patients who exhibit it [4].

Syncope is much easier to recognize than pulmonary embolism; however, it might be difficult to determine the etiology of this symptom. In the given patient, the only definitive link with pulmonary embolism could be established because of the patient's family history pointing towards 49 genetic risk factors of the same. Genetic risk factors need to be considered in cases of Pulmonary Embolism when symptoms are not obvious and diagnosis is difficult [5]. According to the Modified Wells Scoring System, the given patient fulfills only one criterion, i.e., PE is more likely than other diagnoses, thus scoring a 3 on the scoring system, with an intermediate probability of PE. The patient does not fit into any other diagnostic profile for pulmonary embolism, thus making the diagnosis difficult [6].

The current ESC recommendations indicate that individuals with a low or intermediate clinical risk of PE be excluded from PE rather than utilizing a set level of D-dimers (500 ng/mL) [7].

## Conclusions

When syncope is the sole presenting symptom, it may be challenging to diagnose pulmonary embolism. Physicians should keep family history in mind as it might be a useful aid in making a tough PE diagnosis. In order to identify and treat PE, doctors must follow the recent recommendations issued by the European Society of Cardiology and the European Respiratory Society.
